# Visual impairment and all-cause mortality: a real-world retrospective cohort study

**DOI:** 10.3389/fpubh.2025.1670906

**Published:** 2025-11-13

**Authors:** Chenxi Fu, Qinyi Gu, Xi Li, Zhouqian Wang, Xiaoyu Zhang, Wei Chen

**Affiliations:** 1 Ningbo Eye Hospital, Wenzhou Medical University, Ningbo, China; 2 Shanxi Eye Hospital Affiliated to Shanxi Medical University, Taiyuan, Shanxi, China; 3 School of Public Health, Wenzhou Medical University, Wenzhou, China; 4 National Clinical Research Centre for Ocular Diseases, Eye Hospital, Wenzhou Medical University, Wenzhou, China; 5 Ningbo Key Laboratory of Medical Research on Blinding Eye Diseases, Ningbo Eye Institute, Ningbo Eye Hospital, Wenzhou Medical University, Ningbo, China

**Keywords:** visual impairment, all-cause mortality, real-world study, cohort study, survival analysis

## Abstract

**Background:**

VI (visual impairment) significantly impacts global public health, and there are few studies and inconsistent results on the impact of VI on mortality in Chinese adults. Our study aims to investigate the association between VI and the all-cause mortality risk in Chinese adults and to explore potential sex differences.

**Methods:**

This retrospective cohort (from July 17, 2010 to September 30, 2021) utilized data from the Yinzhou Health Information System, involving 182,468 individuals with valid visual acuity (VA) examination records. We assessed VI, defined as a presenting VA of the better eye worse than 0.5, using two variables: a binary variable (non-VI vs. VI) and a categorical variable (non-VI: VA ≥ 0.5; mild VI: 0.3 ≤ VA < 0.5; moderate VI: 0.1 ≤ VA < 0.3; severe VI: VA < 0.1). Cox proportional hazards multivariable regression models were used to estimate the hazard ratios (HRs) and 95% confidence intervals (CIs) for the all-cause mortality risk. Subgroup analyses were also conducted based on sex and age.

**Results:**

During a median follow-up of 3.87 years, there were 2,632 death events, with 1,579 occurring in the non-visual impairment (VI) group and 1,053 in the VI group. Individuals with VI (VA < 0.5) had an increased all-cause mortality risk (HR, 1.15; 95% CI, 1.06–1.24), and this association persisted in old adults (HR, 1.15; 95% CI, 1.06–1.25). Old females with moderate or severe VI showed significantly higher all-cause mortality risks, with HRs of 1.32 (95% CI, 1.13–1.55) and 2.16 (95% CI, 1.35–3.46), respectively. Among old male participants, the increased all-cause mortality risk was only observed in those with moderate VI (HR, 1.42; 95% CI, 1.23–1.65).

**Conclusion:**

VI was associated with an increased all-cause mortality risk in Chinese old population. Sex differences were also observed in the associations between the VI level and the all-cause mortality risk.

## Introduction

Over half a billion to 1.3 billion individuals suffer from visual impairment (VI) or blindness worldwide, and this number is projected to more than double or even triple in the next few decades (1, 2). The impacts of VI are far-reaching, including negative effects on quality of life, heightened risks of falls, and loss of independence (3–5). And VI is associated with increased risks of cognitive impairment, dementia, depression, and mortality (6–14). Consequently, VI imposes a substantial burden on global public health (15, 16).

Cohort and meta-analysis studies have reported the association between VI and an increased risk of all-cause mortality in many populations and countries (14, 17–21). Previous research predominantly comprised cohort studies that involved thousands of participants or relied on self-reported VI rather than objective visual acuity (VA) measurements (17, 18). Moreover, when examining the association between VI and mortality, most previous studies focused on specific age groups, particularly the population above 60 (18–20). Few studies included adults of all ages and explored the differences between the young to middle-aged and older population. From existing studies, conclusions are also inconsistent on whether there is a sex disparity in the mortality risk associated with VI (18, 19, 22–25). Furthermore, there have been limited studies and differing results on the relationship between VI and mortality among Chinese adults (26–29). Therefore, it is necessary to investigate the association between VI and mortality and explore sex differences across a large-scale population of all ages with objective measures of VA.

With the development of integrated electronic health information, large regional health databases enable us to conduct exploratory research across the overall adult population. The Yinzhou Health Information System (YHIS) is a regional health database that was initiated in 2006 by the Yinzhou District Center for Disease Control and Prevention in Ningbo, China. The detailed information about YHIS was described previously (30). It includes sociodemographic information, longitudinal health examination and medical history records, chronic disease management information, and death certificates for nearly 98% of the permanent residents in Yinzhou over a period exceeding 10 years.

To address the gaps in previous research among Chinese adults explore potential sex-based differences, we conducted a retrospective cohort study to examine the relationship between VI and the risk of all-cause mortality using large-scale, real-world data from the YHIS.

## Materials and methods

### Data source and study population

**Figure 1:**
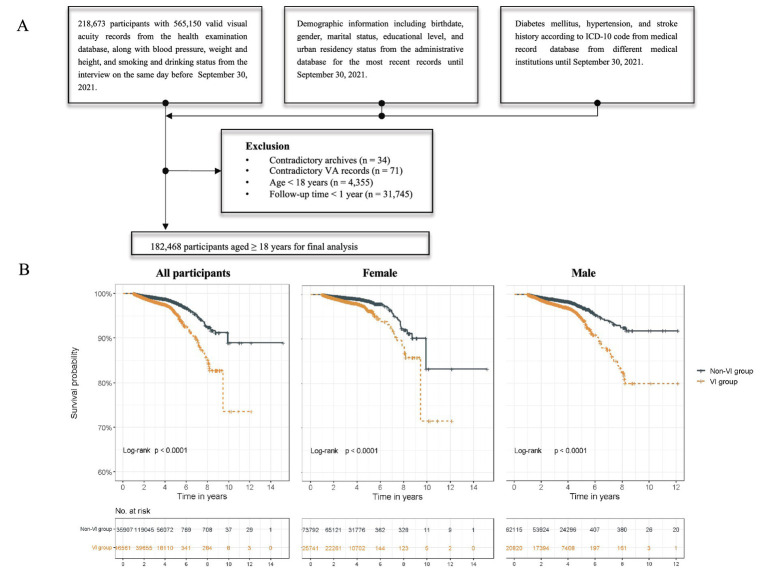
Flowchart and Kaplan–Meier survival curve. **(A)** Flowchart of study participants. **(B)** Survival curves of non-VI and VI groups in all participants, females, and males.

The participants in our study were residents with unique identifiers and VA examination records from the routine health examination from July 17, 2010 to September 30, 2021 in YHIS. Data from administrative records, routine health examination, and medical record databases of YHIS were collected and linked to each individual through an encrypted unique identification number. We excluded subjects with contradictory health archives and VA records (n = 34 and n = 71, respectively), those under 18 years old at the VA examination date (*n* = 4,355), and those who followed for less than 1 year (*n* = 31,745). A total of 182,468 participants were included in the final analysis (Figure 1A).

This study adhered to the principles of the Declaration of Helsinki and was approved by the Ethics Committee of Ningbo Eye Hospital, Wenzhou Medical University (Approval Number: 2023-33(K)-X1). The requirement for informed consent was waived due to the use of anonymous and de-identified registry-based secondary data in our analysis.

### Visual acuity and covariates

Either the uncorrected VA or the corrected VA (with habitual correction) was used as the presenting VA. The presenting VA of the better eye was used for further analysis. VA was recorded in decimal value in YHIS, and invalid values were treated as missing data (VA < 0.01 or VA > 2.0). VI was defined as VA worse than 0.5. VI was further subdivided into mild VI subgroup (0.3 ≤ VA < 0.5), moderate VI subgroup (0.1 ≤ VA < 0.3), and severe VI subgroup (VA < 0.1).

The selection of potential confounders was based on a review of prior studies and the variables available in the YHIS dataset, including known demographic, physical, and clinical factors that could influence the relationship between VI and mortality. Demographic information, including date of birth, sex, marital status, educational level, and urban residency status, was derived from the latest administrative records and reclassified. Information on smoking status (never, quit, and current) and drinking status (never, occasionally, often, everyday) was obtained from the health examination records of the same day as the VA measurement and subsequently reclassified for analysis. Missing values for categories were assigned a separate category (smoking status, 3.61%; drinking status, 3.46%; marital status, 0.85%; educational level, 1.60%; and urban residency status, 0.01%).

Physical examination information on height, weight, systolic blood pressure (SBP), and diastolic blood pressure (DBP) was obtained from the routine health examination on the same day with VA measurement to calculate body mass index (BMI) and mean arterial blood pressure (MABP). Extreme values of variables (height < 140 or > 200 cm, weight < 40 or > 150 kg, SBP > 200 or < 80 mmHg, DBP > 120 or < 50 mmHg) were treated as missing data. BMI was categorized into four groups according to the guidelines for prevention and control of overweight and obesity Chinese adults for further analysis: underweight (BMI < 18.5 kg/m^2^), normal weight (18.5 ≤ BMI < 24.0 kg/m^2^), overweight (24.0 ≤ BMI < 28.0 kg/m^2^), and obesity (BMI ≥ 28.0 kg/m^2^) (31). The missing BMI data (6.20%) was treated as a separate category. Multiple imputation was used to handle missing MABP data (22.8%).

Chronic disease histories (diabetes mellitus, hypertension, and stroke histories) were defined based on outpatient medical records using the International Classification of Diseases, Tenth Revision (ICD-10) codes. Participants were considered to have a history of chronic disease if they had at least one outpatient medical record with the corresponding ICD-10 code during the follow-up period. The ICD-10 diagnosis codes for diabetes mellitus history were the codes beginning with E10, E11, E12, E13, and E14, the ones beginning with I10 for hypertension history, and the ones beginning with I60, I61, I62, I63, I64, and I69 for stroke history. The corresponding diagnostic names for the ICD-10 codes used to define chronic disease histories are provided in Supplementary Table S5.

### Mortality and follow-up

Mortality follow-up was conducted using verified death certificates obtained from hospitals and uploaded to the YHIS. Participants were followed from the date of their initial visual acuity (VA) examination until the occurrence of death or September 30, 2021, whichever event transpired first. Since the participants were permanent residents and the YHIS has broad coverage, loss to follow-up was not considered.

### Statistical analysis

Participant characteristics were summarized utilizing descriptive statistics. Continuous variables were presented as mean ± standard deviation (SD) or median (interquartile range, IQR), depending on the distribution’s normality, which was evaluated using Q-Q plots. Categorical variables were presented as frequencies and percentages (n, %). The standardized mean difference (SMD) was used to compare variables between the non-VI group and the VI group, with an SMD exceeding 0.1 indicating a significant difference in variables. The mortality rate was calculated by dividing the number of death events by the total follow-up person-years (PYs).

The Kaplan–Meier survival analyses with a log-rank test were used to compare the risks of all-cause mortality between participants with and without VI in all participants, as well as in female and male participants. Cox proportional hazard regression was employed to estimate the hazard ratios (HRs) and 95% confidence intervals (CIs) for all-cause mortality in the VI group, with the non-VI group serving as the reference. Model 1 was adjusted for age and sex, while model 2 was further adjusted for marital status, educational level, urban residency status, smoking and drinking status, diabetes mellitus history, hypertension history, stroke history, BMI category, and MABP based on model 2. The assumption of proportional hazards was evaluated by statistical tests based on weighted Schoenfeld residuals (32), and no variables were found to violate this assumption. We first investigated the association between VI (with multiple VI levels) and mortality in the overall population, as well as separately in old (≥ 60 years) and young to middle-aged (< 60 years) participants and in female and male participants. Subsequently, subgroup analyses were conducted to explore associations with sex and age stratification. In a sensitivity analysis, we excluded participants with less than 2 years of follow-up to ensure the robustness of our main results. The analyses were then conducted on this subgroup, with stratifications by overall, sex, and age group and sex (*n* = 158,700).

All statistical analyses were performed utilizing R software (version 4.2.3)^1^. All statistical tests were two-sided, and a *p* value below 0.05 was considered statistically significant.

## Results

### Baseline characteristics

Among the 182,468 participants included in the final analysis, 46,561 individuals (25.52%) exhibited VI. The mean age of all participants was 60.95 ± 13.40 years, ranging from 18.00 to 101.60 years, and 54.55% were female. Table 1 presents the characteristics of participants categorized by VI status. Participants with VI tended to be older, current smokers, current drinkers, and have higher SBP. The characteristics of 158,700 participants in the sensitivity analysis are detailed in Supplementary Table S1.

**Table 1:** Characteristics of participants stratified by visual acuity category.

	Overall	Non-VI group	VI group	SMD
	(*n* = 182,468)	(*n* = 135,907)	(*n* = 46,561)	
Age (year), mean ± SD	60.95 (13.40)	59.43 (13.53)	65.42 (11.91)	0.470
Follow-up time (year), median [IQR]	3.87 [2.77, 4.24]	3.87 [2.78, 4.25]	3.82 [2.76, 4.23]	0.059
Female, *n* (%)	99,533 (54.55)	73,792 (54.30)	2,5,741 (55.28)	0.020
Marriage status, *n* (%)				
Unmarried	16,245 (8.90)	11,173 (8.22)	5,072 (10.89)	0.092
Married	164,671 (90.25)	123,616 (90.96)	41,055 (88.17)	
Education level, *n* (%)				
Less than junior secondary	141,822 (77.72)	103,549 (76.19)	38,273 (82.2)	0.149
More than junior secondary	37,720 (20.67)	30,097 (22.15)	7,623 (16.37)	
Urban residency status (%)				
Rural	90,377 (49.53)	65,782 (48.40)	24,595 (52.82)	0.089
Urban	92,072 (50.46)	70,115 (51.59)	21,957 (47.16)	
Drink status, *n* (%)				
Never or quit	139,711 (76.57)	103,407 (76.09)	36,304 (77.97)	0.137
Current drinker	36,450 (19.98)	27,008 (19.87)	9,442 (20.28)	
Smoke status, *n* (%)				
Never or quit	147,459 (80.81)	109,060 (80.25)	38,399 (82.47)	0.137
Current smoker	28,424 (15.58)	21,132 (15.55)	7,292 (15.66)	
BMI categories, *n* (%)				
Normal weight	93,696 (51.35)	70,033 (51.53)	23,663 (50.82)	0.046
Underweight	6,367 (3.49)	4,757 (3.50)	1,610 (3.46)	
Overweight	57,642 (31.59)	42,527 (31.29)	15,115 (32.46)	
Obesity	13,444 (7.37)	9,856 (7.25)	3,588 (7.71)	
Diabetes history, *n* (%)	16,397 (8.99)	11,698 (8.61)	4,699 (10.09)	0.051
Hypertension history, *n* (%)	47,627 (26.10)	34,254 (25.20)	13,373 (28.72)	0.079
Stroke history, *n* (%)	7,093 (3.89)	4,835 (3.56)	2,258 (4.85)	0.064
BMI (kg/m2), mean ± SD	23.55 (3.06)	23.53 (3.06)	23.61 (3.07)	0.026
SBP (mmHg), mean ± SD	131.19 (16.89)	130.49 (17.05)	133.15 (16.27)	0.160
DBP (mmHg), median ± SD	78.36 (9.54)	78.53 (9.70)	77.88 (9.06)	0.070
MABP (mmHg), mean ± SD	95.89 (10.37)	95.76 (10.56)	96.25 (9.82)	0.049

Visual acuity categories were categorized according to presenting VA of the better eye (non-VI group, VA ≥ 0.5; VI group, VA < 0.5).

Data were reported as mean ± SD or median [IQR] for continuous variables and number (proportion) for categorical variables.

SMD was used to compare the variables between non-VI group and VI group.

VI, visual impairment; VA, visual acuity; SMD, standard mean difference; SD, standard deviation; IQR, interquartile range; BMI, body mass index; SBP, systolic blood pressure; DBP, diastolic blood pressure; MABP, mean arterial blood pressure.

### Visual impairment and mortality

During a median follow-up of 3.87 years (IQR, 2.77–4.24), there were 2,632 death events among all participants. Participants with VI had a mortality rate more than twice that of those without VI (660.76 versus 332.87 per 10,000 PY, respectively). The mortality rate increased with the severity of VI in all participants, and the same results were observed in the age stratification subgroups. Participants with a VA lower than 0.5 (VI group) had a higher relative risk for all-cause mortality, with an adjusted HR of 1.15 (95% CI, 1.06–1.24) in model 2 (Table 2). The Kaplan–Meier survival curve revealed significantly lower survival probabilities in individuals with VI compared to those without VI among all participants. Subgroup analysis, stratified by age, indicated that the increased mortality risk associated with VI was only observed in participants aged over 60 years, with an adjusted HR of 1.15 (95% CI, 1.06–1.25). Similar results were observed when categorizing VI into severity levels. Participants with severe VI exhibited a notably higher mortality risk (adjusted HR, 1.66; 95% CI, 1.22–2.27) compared to the moderate VI group (adjusted HR, 1.37; 95% CI, 1.23–1.52), with a dose–response relationship observed (*p* for trend < 0.001). Among the adults 60 and above, the adjusted HR was 1.37 (95% CI, 1.23–1.53) in the moderate VI group and 1.61 (95% CI, 1.16–2.22) in the severe VI group. These associations and trends observed in the overall and old participants were not found in the young to middle-aged participants (age < 60 years).

**Table 2:** Association between visual impairment and mortality.

	VA category	Follow-up duration, PY	Event/Total, n	Mortality rate, 10^5^ PY	Model 1		Model 2	
					HR (95% CI)	*p* value	HR (95% CI)	*p* value
All (*n* = 182,468)	Non-VI group	474,354.91	1,579/135,907	332.87	Reference	Reference		
	VI group	159,363.07	1,053/46,561	660.76	1.13 (1.05–1.23)	0.002	1.15 (1.06–1.24)	< 0.001
	Mild VI subgroup	104,227.23	535/31,204	513.30	0.97 (0.88–1.07)	0.540	0.98 (0.89–1.09)	0.738
	Moderate VI subgroup	51,179.83	477/14,286	932.01	1.36 (1.22–1.51)	< 0.001	1.37 (1.23–1.52)	< 0.001
	Severe VI subgroup	3,956.01	41/1,071	1,036.40	1.73 (1.27–2.36)	< 0.001	1.66 (1.22–2.27)	0.001
	*p* for trend				< 0.001		< 0.001	
Old (age ≥ 60 years) (*n* = 109,777)	Non-VI group	271,162.88	1,439/76,609	530.68	Reference	Reference		
	VI group	114,861.32	1,019/33,168	887.16	1.14 (1.05–1.23)	0.002	1.15 (1.06–1.25)	0.001
	Mild VI subgroup	73,629.22	514/2,1781	698.09	0.97 (0.88–1.08)	0.607	0.99 (0.89–1.09)	0.773
	Moderate VI subgroup	38,511.84	467/10,681	1,212.61	1.36 (1.22–1.51)	< 0.001	1.37 (1.23–1.53)	< 0.001
	Severe VI subgroup	2,720.25	38/706	1,396.93	1.67 (1.21–2.31)	0.002	1.61 (1.16–2.22)	0.004
	*p* for trend				< 0.001		< 0.001	
Young to middle-aged (age < 60 years) (*n* = 72,691)	Non-VI group	203,192.04	140/59,298	68.90	Reference	Reference		
	VI group	44,501.75	34/13,393	76.40	0.91 (0.62–1.32)	0.617	0.96 (0.66–1.40)	0.831
	Mild VI subgroup	30,598.01	21/9,423	68.63	0.81 (0.51–1.28)	0.363	0.87 (0.55–1.39)	0.563
	Moderate VI subgroup	12,667.99	10/3,605	78.94	0.97 (0.51–1.84)	0.918	0.98 (0.51–1.86)	0.942
	Severe VI subgroup	1,235.75	3/365	242.77	2.76 (0.88–8.66)	0.083	2.58 (0.82–8.13)	0.107
	*p* for trend				0.894		0.758	

Model 1: Adjusted for age and sex.

Model 2: Adjusted for age, sex, marital status, educational level, urban residency status, smoking status, drinking status, diabetes mellitus history, hypertension history, stroke history, BMI category, and MABP.

VA categories were categorized according to presenting VA of the better eye (non-VI group, VA ≥ 0.5; VI group, VA < 0.5; mild VI subgroup, 0.3 ≤ VA < 0.5; moderate VI subgroup, 0.1 ≤ VA < 0.3; severe VI subgroup, VA < 0.1).

VI, visual impairment; VA, visual acuity; PY, person-year; HR, hazard ratio; CI, confidence interval; BMI, body mass index; MABP, mean arterial blood pressure.

In the sex-stratified analysis, different results were observed among male and female participants (Table 3). A heightened mortality risk associated with VI was found in male participants (HR, 1.18; 95% CI, 1.06–1.31) but not in female participants. The survival curves indicated that males without VI had a higher survival probability compared to those with VI (Figure 1B). When examining various levels of VI severity, an increased mortality risk was observed in females with moderate VI (HR, 1.30; 95% CI, 1.11–1.52) and severe VI (HR, 2.14; 95% CI, 1.35–3.39); while the increased risk was only noted in males with moderate VI (HR, 1.44; 95% CI, 1.25–1.66) but not in those with severe VI. Additionally, the increased mortality risk associated with VI was not found in the mild VI subgroup for overall, female, and male participants.

**Table 3:** Association between visual impairment and mortality in female and male participants.

	VA category	Follow-up duration, PY	Event/Total, *n*	Mortality rate, 10^5^ PY	Model 1		Model 2	
					HR (95% CI)	*p* value	HR (95% CI)	*p* value
Female (*n* = 99,533)	Non-VI group	259,457.29	644/73,792	248.21	Reference		Reference	
	VI group	89,109.55	480/25,741	538.66	1.09 (0.97–1.23)	0.154	1.12 (1.00–1.27)	0.059
	Mild VI subgroup	56,911.73	239/16,841	419.95	0.93 (0.80–1.08)	0.370	0.97 (0.83–1.12)	0.664
	Moderate VI subgroup	30,220.60	222/8,349	734.60	1.27 (1.09–1.49)	0.002	1.30 (1.11–1.52)	0.001
	Severe VI subgroup	1,977.22	19/551	960.95	2.22 (1.41–3.51)	< 0.001	2.14 (1.35–3.39)	0.001
	*p* for trend				1.13 (1.05–1.21)	0.001	1.14 (1.06–1.23)	< 0.001
Male (*n* = 82,935)	Non-VI group	214,897.62	935/62,115	435.09	Reference		Reference	
	VI group	70,253.52	573/20,820	815.62	1.17 (1.05–1.30)	0.0041	1.18 (1.06–1.31)	0.003
	Mild VI subgroup	47,315.50	296/14,363	625.59	1.00 (0.87–1.14)	0.9672	1.01 (0.88–1.15)	0.914
	Moderate VI subgroup	20,959.24	255/5,937	1,216.65	1.43 (1.25–1.65)	0.0000	1.44 (1.25–1.66)	< 0.001
	Severe VI subgroup	1,978.79	22/520	1,111.79	1.48 (0.97–2.26)	0.0697	1.42 (0.93–2.17)	0.107
	*p* for trend				1.16 (1.09–1.24)	< 0.001	1.16 (1.09–1.24)	< 0.001

Model 1: Adjusted for age and sex.

Model 2: Adjusted for age, sex, marital status, educational level, urban residency status, smoking status, drinking status, diabetes mellitus history, hypertension history, stroke history, BMI category, and MABP.

VA categories were categorized according to presenting VA of the better eye (non-VI group, VA ≥ 0.5, VI group, VA < 0.5; mild VI subgroup, 0.3 ≤ VA < 0.5; moderate VI subgroup, 0.1 ≤ VA < 0.3; severe VI subgroup, VA < 0.1).

VI, visual impairment; VA, visual acuity; PY, person-year; HR, hazard ratio; CI, confidence interval; BMI, body mass index; MABP, mean arterial blood pressure.

Given that the associations between VI and increased mortality risk were primarily found in the older population, further analyses were conducted in subgroups stratified by age and sex. The analyses revealed similar sex-specific differences at multiple levels of VI in old and the entire participant (Tables 1, 4). A significant adjusted HR (HR, 1.15; 95% CI, 1.01–1.30) was observed in old females with VI, which was not observed in the overall female population with VI. The insignificant associations with wide CIs observed in the young to middle-aged participants, particularly within the severe VI subgroup, were likely due to the limited number of death events.

**Table 4:** Association between visual impairment and mortality in female and male stratified by age.

		VA category	Follow-up duration, PY	Event/Total, *n*	Mortality rate, 10^5^ PY	Model 1		Model 2	
						HR (95% CI)	*p* value	HR (95% CI)	*p* value
Old (≥ 60 years)	Female (*n* = 56,447)	Non-VI group	137,236.30	581/38,566	423.36	Reference		Reference	
		VI group	62,463.42	469/17,881	750.84	1.11 (0.98–1.26)	0.088	1.15 (1.01–1.30)	0.030
		Mild VI subgroup	39,379.61	232/11,550	589.14	0.95 (0.82–1.11)	0.551	0.99 (0.85–1.15)	0.888
		Moderate VI subgroup	21,815.73	219/5,991	1,003.86	1.29 (1.10–1.51)	0.002	1.32 (1.13–1.55)	< 0.001
		Severe VI subgroup	1,268.08	18/340	1,419.47	2.23 (1.40–3.57)	< 0.001	2.16 (1.35–3.46)	0.001
		*p* for trend				< 0.001		< 0.001	
	Male (*n* = 53,330)	Non-VI group	133,926.58	858/38,043	640.65	Reference		Reference	
		VI group	52,397.90	550/15,287	1,049.66	1.16 (1.04–1.29)	0.008	1.16 (1.04–1.30)	0.007
		Mild VI subgroup	34,249.62	282/10,231	823.37	0.99 (0.87–1.14)	0.899	1.00 (0.87–1.14)	0.969
		Moderate VI subgroup	16,696.11	248/4,690	1,485.38	1.42 (1.23–1.64)	< 0.001	1.42 (1.23–1.65)	< 0.001
		Severe VI subgroup	1,452.18	20/366	1,377.24	1.39 (0.89–2.17)	0.145	1.33 (0.85–2.08)	0.209
		*p* for trend				< 0.001		< 0.001	
Young to middle-aged (< 60 years)	Female (*n* = 43,086)	Non-VI group	122,220.99	63/35,226	51.55	Reference		Reference	
		VI group	26,646.13	11/7,860	41.28	0.63 (0.33–1.20)	0.159	0.65 (0.34–1.24)	0.189
		Mild VI subgroup	17,532.13	7/5,291	39.93	0.61 (0.28–1.32)	0.209	0.63 (0.29–1.39)	0.256
		Moderate VI subgroup	8,404.86	3/2,358	35.69	0.55 (0.17–1.75)	0.312	0.55 (0.17–1.76)	0.314
		Severe VI subgroup	709.14	1/211	141.02	2.25 (0.31–16.21)	0.422	2.09 (0.29–15.15)	0.466
		*p* for trend				0.270		0.296	
	Male (*n* = 29,605)	Non-VI group	80,971.05	77/24,072	95.10	Reference		Reference	
		VI group	17,855.62	23/5,533	128.81	1.15 (0.72–1.83)	0.560	1.22 (0.76–1.96)	0.401
		Mild VI subgroup	13,065.88	14/4,132	107.15	0.97 (0.55–1.72)	0.923	1.07 (0.60–1.91)	0.815
		Moderate VI subgroup	4,263.13	7/1,247	164.20	1.41 (0.65–3.06)	0.387	1.39 (0.63–3.02)	0.413
		Severe VI subgroup	526.61	2/154	379.79	3.12 (0.77–12.74)	0.112	2.80 (0.68–11.53)	0.154
		*p* for trend				0.237		0.200	

Model 1: Adjusted for age and sex. Model 2: Adjusted for age, sex, marital status, educational level, urban residency status, smoking status, drinking status, diabetes mellitus history, hypertension history, stroke history, BMI category, and MABP. VA categories were categorized according to presenting VA of the better eye (non-VI group, VA ≥ 0.5, VI group, VA < 0.5; mild VI subgroup, 0.3 ≤ VA < 0.5; moderate VI subgroup, 0.1 ≤ VA < 0.3; severe VI subgroup, VA < 0.1).

VI, visual impairment; VA, visual acuity; PY, person-year; HR, hazard ratio; CI, confidence interval; BMI, body mass index; MABP, mean arterial blood pressure.

### Sensitivity analysis

Sensitivity analysis revealed consistent findings among participants with follow-up time longer than 2 years (refer to Supplementary Tables S2–S4). In addition, the increased mortality risk associated with VI was observed in the VI group in females across all age groups, as well as in severe VI subgroups of young to middle-aged overall and male participants.

## Discussion

### Findings and comparison

In this large retrospective cohort study involving 182,468 Chinese adults, individuals with VI (VA < 0.5) showed a higher risk of all-cause mortality compared to those without VI among the old population (aged ≥ 60 years). Additionally, the association between VI and increased mortality risk was not found in participants with mild VI (0.3 ≤ VA < 0.5) but in participants with worse VA (moderate to severe VI, VA < 0.3). Furthermore, the associations varied between sexes at different levels of VI. Females with moderate to severe VI (VA < 0.3) faced a heightened risk of all-cause mortality, which increased with the severity of VI. However, males with moderate VI (0.1 ≤ VA < 0.3) had an increased risk of all-cause mortality, whereas those with severe VI did not demonstrate a heightened risk compared to males without VI.

The relationship between VI and mortality has been extensively explored worldwide. Previous studies predominantly focused on specific age groups, particularly the old population, and have involved thousands of participants. These studies were constrained by limitations in design and cost inherent in cohort studies. Our study, conducted on a large scale with over 180,000 adults of all ages, utilized real-world data facilitated by the widespread adoption of electronic health records technology, thus encompassing a wide demographic range. A meta-analysis incorporating 30 cohorts worldwide, comprising 446,088 participants revealed that individuals with VI (VA < 6/12) faced an elevated risk of all-cause mortality, with a pooled HR of 1.29 (95% CI, 1.20–1.39) (14). Our study observed an increased risk of all-cause mortality among Chinese participants 60 and above with VI (VA < 0.5), with an adjusted HR of 1.15 (95% CI, 1.06–1.25; *p* = 0.001). A recent study involving over 580,000 young to middle-aged individuals in Korea found that VI was associated with increased risks of all-cause and cardiovascular disease-related mortality (21). However, our study did not find this association in Chinese participants under 60 years. Nonetheless, in young to middle-aged participants with a follow-up period exceeding 2 years (refer to Supplementary Tables S2, S4), an increased mortality risk in severe VI group was observed, albeit with a limited number of follow-up individuals and events. Further investigation into the association between VI and the risk of all-cause mortality in young to middle-aged populations is warranted, particularly with an extended follow-up period in the future.

Existing studies have indicated that the association between VI and an increased mortality risk can be attributed to psychological, physical, and psychosocial factors. VI has been reported to be associated with mental well-being issues such as cognitive decline (6, 7), possible cognitive impairment (8), and depression (13). Furthermore, it has been linked to psychosocial conditions that affect mental well-being, such as social isolation (33), loss of independence (34), and diminished social interaction (35). Individuals with VI may experience barriers to maintain healthy lifestyles including regular exercise, which might consequently lead to increased risk of mortality (36, 37). Mediating pathways between VI and mortality, which could include shared risk factors, such as physical inactivity, social isolation, and disability. In the study of Karpa et al. (38), disability in walking was a major indirect factor linking between VI and mortality, aligning findings on the impact of daily living activities (39). Few studies have reported the associations between mild VI (0.3 ≤ VA < 0.5) and all-cause mortality, using a non-VI group with VA of ≥ 0.5 as a reference. In our study, participants with mild VI did not exhibit an increased risk of all-cause mortality compared to those without VI, possibly due to the minor impact of mild VI on daily activities. In addition to these pathways, there may be shared biological risk factors. Some causes of VI, such as cataract, glaucoma, and age-related macular degeneration, are often considered markers of aging and may suggest accelerated biological aging (40). Furthermore, systemic diseases like hypertension and diabetes can directly cause ocular complications leading to VI (24). It’s plausible that these underlying diseases and shared risk factors act as mediating pathways between VI and mortality.

Several studies have investigated the associations between VI and the risk of all-cause mortality across sexes using either objective VA or self-reported VI, yielding inconclusive results. Studies using objective VA did not consistently find a link between VI and higher mortality risk in either males or females (22, 23), though one study did identify such an association in females (24). However, studies using self-reported VI found a clear link between VI and higher mortality in both sexes (18, 19, 25), with severe bilateral VI associated with higher mortality only in females (25). The variability in outcomes can be attributed to various factors, including the age range of study subjects, VI definitions, ethnic backgrounds, analytical methods, and gender-based societal roles across studies. In our study, the association between VI and increased all-cause mortality was observed only in the overall male population and in both sexes among old participants. Among the old population, VI was associated with declines in cognitive function, physical activity, and social engagement, with these effects being more pronounced in males than in females (8, 41). This discrepancy may elucidate the slightly higher risks of mortality observed in old males compared to females at the same levels of VI in our study. However, for severe VI (VA < 0.1), our study found an increased risk for all-cause mortality only in old females but not males. According to the World Health Organization report, the life expectancy at age 60 in China in 2019 was 19.2 years for males and 23.1 years for females (42), which might be attributed to a higher proportion of old females living alone compared to males (43). Additionally, due to cultural gender roles differing in China, old males with severe VI are more likely to have a supporting partner for daily life and medical care, which might mitigate the impact of VI on mortality.

### Implications

Despite the complex and not entirely comprehended mechanisms linking VI and all-cause mortality, the findings from our study offer significant implications for public health and clinical practice. Firstly, the evident association between VI and a higher mortality risk underscores the necessity for public health strategies focused on screening and preventing ocular diseases leading to irreversible vision loss. It is estimated that up to 80% of cases of VI and blindness are preventable or treatable. Cataracts and uncorrected refractive errors are the predominant causes of VI and blindness worldwide (44). These conditions can be effectively addressed with cost-effective, low-cost interventions, such as cataract surgery and the provision of inexpensive, easily produced lenses. Secondly, improving VA in clinical practice would contribute to reducing individual mortality risk and alleviating the public health burden. Based on our results, the independent effect of VI on all-cause mortality could be mitigated by improving presenting VA above 0.3. Thirdly, there is a need for public and social organizations to establish follow-up systems to provide support for old individuals with moderate to severe VI (VA < 0.3), particularly for old females experiencing severe VI (VA < 0.1).

### Strengths and limitations

To our knowledge, this study is among the few that utilized real-world evidence to investigate the association between VI and risk of all-cause mortality. The availability of extensive real-world follow-up data from multiple sources has enabled the exploration of association in a large cohort, including adults of all ages, incorporating the authenticity and updates of potential confounding factors during the study period, such as medical histories obtained from healthcare institutions. Furthermore, our study explored the associations across various age groups (old and young to middle-aged subgroups), sex stratification, and VI severity levels by employing multivariable models and sensitivity analysis.

Nevertheless, several limitations of our study warrant mention. Firstly, the VA data from different health examination institutions may contain errors due to variations in testing environments. Despite this, the measurement of VA is highly standardized and easily implementable in practical settings. Secondly, the association was explored over a relatively short follow-up period due to the data update schedule of YHIS, resulting in a limited number of events in subgroup analyses, especially among young to middle-aged participants. Thirdly, time-dependent analyses with confounders as time-varying covariates were not conducted due to the constrained follow-up duration. We plan to reassess the association between VI and mortality risk with an extended follow-up period using YHIS data in the future. Fourthly, our study utilized data from a specific area in China, limiting its generalizability to other countries and populations. Further studies using real-world data across diverse regions are needed. Fifthly, the analyses of cause-specific mortality, cardiovascular-related, injury-related, or cancer-related mortality for example, were not conducted due to the lack of detailed information on specific causes of death, which need further research. Sixthly, some potential confounders, such as socioeconomic status (personal income) and physical activity, were not adjusted for. These unmeasured factors, inherent in our real-world data system, might have influenced the observed associations. Lastly, since this study was conducted among residents with VA examination records, the generalizability of our findings may be limited. Nevertheless, the participants and VA data in our study were from routine health examination records but not from outpatient records, which made our study population have a good representativeness.

In summary, our study revealed that VI was associated with an increased risk of all-cause mortality in the Chinese old participants 60 and above. Furthermore, the degree of VI had a differential impact on the risk of all-cause mortality among Chinese males and females.

## Data Availability

The data analyzed in this study is subject to the following licenses/restrictions: the data that support the findings of this study are available from Yinzhou District Center for Disease Control and Prevention, Ningbo, China, but restrictions apply to the availability of these data, which were used under license for the current study, and so are not publicly available. Data are however available from the authors upon reasonable request and with permission of Yinzhou District Center for Disease Control and Prevention. Requests to access these datasets should be directed to Chenxi Fu, fuchenxi7302@gmail.com.

## Footnotes

^1^ https://www.R-project.org/

## References

[1] Bourne RRA Flaxman SR Braithwaite T Cicinelli MV Das A Jonas JB . Magnitude, temporal trends, and projections of the global prevalence of blindness and distance and near vision impairment: a systematic review and meta-analysis. Lancet Glob Health. (2017) 5:e888–97. doi: 10.1016/S2214-109X(17)30293-0, PMID: 28779882

[2] Bourne R Steinmetz JD Flaxman S Briant PS Taylor HR Resnikoff S . Trends in prevalence of blindness and distance and near vision impairment over 30 years: an analysis for the global burden of disease study. Lancet Glob Health. (2021) 9:e130–43. doi: 10.1016/S2214-109X(20)30425-3, PMID: 33275950 PMC7820390

[3] Varma R Wu J Chong K Azen SP Hays RD. Impact of severity and bilaterality of visual impairment on health-related quality of life. Ophthalmology. (2006) 113:1846–53. doi: 10.1016/j.ophtha.2006.04.028, PMID: 16889831

[4] Lopez D McCaul KA Hankey GJ Norman PE Almeida OP Dobson AJ . Falls, injuries from falls, health related quality of life and mortality in older adults with vision and hearing impairment—is there a gender difference? Maturitas. (2011) 69:359–64. doi: 10.1016/j.maturitas.2011.05.006, PMID: 21664773

[5] Singh RR Maurya P. Visual impairment and falls among older adults and elderly: evidence from longitudinal study of ageing in India. BMC Public Health. (2022) 22:2324. doi: 10.1186/s12889-022-14697-2, PMID: 36510173 PMC9746100

[6] Lin MY Gutierrez PR Stone KL Yaffe K Ensrud KE Fink HA . Vision impairment and combined vision and hearing impairment predict cognitive and functional decline in older women. J Am Geriatr Soc. (2004) 52:1996–2002. doi: 10.1111/j.1532-5415.2004.52554.x, PMID: 15571533

[7] De La Fuente J Hjelmborg J Wod M de la Torre-Luque A Caballero FF Christensen K . Longitudinal associations of sensory and cognitive functioning: a structural equation modeling approach. J Gerontol B Psychol Sci Soc Sci. (2019) 74:1308–16. doi: 10.1093/geronb/gby147, PMID: 30521005

[8] Wang P Wang Z Liu X Zhu Y Wang J Liu J. Gender differences in the association between sensory function and CIND among Chinese elderly: based on CLHLS. Arch Gerontol Geriatr. (2023) 113:105054. doi: 10.1016/j.archger.2023.105054, PMID: 37210874

[9] Luo Y He P Guo C Chen G Li N Zheng X. Association between sensory impairment and dementia in older adults: evidence from China. J Am Geriatr Soc. (2018) 66:480–6. doi: 10.1111/jgs.15202, PMID: 29319875

[10] Zhu Z Shi D Liao H Ha J Shang X Huang Y . Visual impairment and risk of dementia: the UK biobank study. Am J Ophthalmol. (2022) 235:7–14. doi: 10.1016/j.ajo.2021.08.010, PMID: 34433084

[11] Littlejohns TJ Hayat S Luben R Brayne C Conroy M Foster PJ . Visual impairment and risk of dementia in 2 population-based prospective cohorts: UK biobank and EPIC-norfolk. Le Couteur D, ed. J Gerontol A Biol Sci Med Sci. (2022) 77:697–704. doi: 10.1093/gerona/glab325, PMID: 34718565 PMC8974347

[12] Zhao X Liu W Lu B Zhu X Zhou M Sun X. Visual impairment and depression in China: a 7-year follow-up study from national longitudinal surveys. BMJ Open. (2022) 12:e055563. doi: 10.1136/bmjopen-2021-055563, PMID: 35477885 PMC9047878

[13] Tournier M Moride Y Ducruet T Moshyk A Rochon S. Depression and mortality in the visually-impaired, community-dwelling, elderly population of Quebec. Acta Ophthalmol. (2008) 86:196–201. doi: 10.1111/j.1600-0420.2007.01024.x, PMID: 17888085

[14] Ehrlich JR Ramke J Macleod D Burn H Lee CN Zhang JH . Association between vision impairment and mortality: a systematic review and meta-analysis. Lancet Glob Health. (2021) 9:e418–30. doi: 10.1016/S2214-109X(20)30549-0, PMID: 33607015 PMC7966688

[15] World Health Organization. World report on Vision. Geneva: World Health Organization (2019).

[16] Committee on Public Health Approaches to Reduce Vision Impairment and Promote Eye Health, Board on Population Health and Public Health Practice, Health and Medicine Division, National Academies of Sciences, Engineering, and Medicine. Making eye health a population health imperative: Vision for tomorrow. New York: National Academies Press (2016).

[17] Zhang T Jiang W Song X Zhang D. The association between visual impairment and the risk of mortality: a meta-analysis of prospective studies. J Epidemiol Community Health. (2016) 70:836–42. doi: 10.1136/jech-2016-207331, PMID: 27095181

[18] Wang Z Congdon N Ma X. Longitudinal associations between self-reported vision impairment and all-cause mortality: a nationally representative cohort study among older Chinese adults. Br J Ophthalmol. (2023) 107:321577. doi: 10.1136/bjo-2022-321577, PMID: 35985659 PMC10646848

[19] ZhangY GeM ZhaoW LiuY XiaX HouL . Sensory impairment and all-cause mortality among the oldest-old: findings from the Chinese longitudinal healthy longevity survey (CLHLS). J Nutr Health Aging. (2020) 24:132–7. doi: 10.1007/s12603-020-1319-2, PMID: 32003401

[20] Wang L Zhu Z Scheetz J He M. Visual impairment and ten-year mortality: the Liwan eye study. Eye. (2021) 35:2173–9. doi: 10.1038/s41433-020-01226-x, PMID: 33077908 PMC8302561

[21] Han SY Chang Y Shin H Choi CY Ryu S. Visual acuity and risk of overall, injury-related, and cardiovascular mortality: the kangbuk samsung health study. Eur J Prev Cardiol. (2022) 29:904–12. doi: 10.1093/eurjpc/zwab025, PMID: 33615358

[22] Gupta S Anand K Agrawal N Kalaivani M Misra P Pandav C. Association of blindness and hearing impairment with mortality in a cohort of elderly persons in a rural area. Indian J Community Med. (2011) 36:208. doi: 10.4103/0970-0218.86522, PMID: 22090675 PMC3214446

[23] Fisher D Li CM Chiu MS Themann CL Petersen H Jonasson F . Impairments in hearing and vision impact on mortality in older people: the AGES-Reykjavik study. Age Ageing. (2014) 43:69–76. doi: 10.1093/ageing/aft122, PMID: 23996030 PMC3861337

[24] Freeman EE Egleston BL West SK Bandeen-Roche K Rubin G. Visual acuity change and mortality in older adults. Invest Ophthalmol Vis Sci. (2005) 46:4040–5. doi: 10.1167/iovs.05-0687, PMID: 16249478

[25] Lee DJ. Visual acuity impairment and mortality in US adults. Arch Ophthalmol. (2002) 120:1544–50. doi: 10.1001/archopht.120.11.1544, PMID: 12427070

[26] Grzybowski A Sak J. The historical development of the concept of glaucoma. Acta Ophthalmol. (2012) 90:e494–6. doi: 10.1111/j.1755-3768.2011.02306.x 22103630

[27] Xu L Wang YX Wang J Jonas JJ. Mortality and ocular diseases. Ophthalmology. (2009) 116:732–8. doi: 10.1016/j.ophtha.2008.11.003, PMID: 19195709

[28] Gu D Zhou J Yong V Sautter J Saito Y. Age differential effects of severity of visual impairment on mortality among older adults in China. J Appl Gerontol. (2013) 32:876–88. doi: 10.1177/0733464812438634, PMID: 25474802 PMC4349516

[29] Li Z Sun D Liu P Zhang L Bai J Cui H. Visual impairment and mortality in a rural adult population (the southern Harbin eye study). Ophthalmic Epidemiol. (2011) 18:54–60. doi: 10.3109/09286586.2010.545503, PMID: 21401412

[30] Lin H Tang X Shen P Zhang D Wu J Zhang J . Using big data to improve cardiovascular care and outcomes in China: a protocol for the CHinese electronic health records research in Yinzhou (CHERRY) study. BMJ Open. (2018) 8:e019698. doi: 10.1136/bmjopen-2017-019698, PMID: 29440217 PMC5829949

[31] Chen C Lu FC. The guidelines for prevention and control of overweight and obesity in Chinese adults. Biomed Environ Sci. (2004) 17:1–36. PMID: 15807475

[32] Grambsch PM Therneau TM. Proportional hazards tests and diagnostics based on weighted residuals. Biometrika. (1994) 81:515–26. doi: 10.1093/biomet/81.3.515

[33] Carabellese C Appollonio I Rozzini R Bianchetti A Frisoni GB Frattola L . Sensory impairment and quality of life in a community elderly population. J Am Geriatr Soc. (1993) 41:401–7. doi: 10.1111/j.1532-5415.1993.tb06948.x, PMID: 8463527

[34] Salive ME Guralnik J Glynn RJ Christen W Wallace RB Ostfeld AM. Association of visual impairment with mobility and physical function. J Am Geriatr Soc. (1994) 42:287–92. doi: 10.1111/j.1532-5415.1994.tb01753.x, PMID: 8120313

[35] Tolman J Hill RD Kleinschmidt JJ Gregg CH. Psychosocial adaptation to visual impairment and its relationship to depressive affect in older adults with age-related macular degeneration. Gerontologist. (2005) 45:747–53. doi: 10.1093/geront/45.6.747, PMID: 16326656

[36] Kim KN Park SJ Kim W Joo J Kim H Kim KH . Modification of the association between visual impairment and mortality by physical activity: a cohort study among the korean national health examinees. Int J Environ Res Public Health. (2019) 16:4386. doi: 10.3390/ijerph16224386, PMID: 31717624 PMC6888179

[37] Smith L Jackson SE Pardhan S López-Sánchez GF Hu L Cao C . Visual impairment and objectively measured physical activity and sedentary behaviour in US adolescents and adults: a cross-sectional study. BMJ Open. (2019) 9:e027267. doi: 10.1136/bmjopen-2018-027267, PMID: 30987991 PMC6500295

[38] Karpa MJ. Direct and indirect effects of visual impairment on mortality risk in older persons. Arch Ophthalmol. (2009) 127:1347. doi: 10.1001/archophthalmol.2009.240, PMID: 19822852

[39] Christ SL Zheng DD Swenor BK Lam BL West SK Tannenbaum SL . Longitudinal relationships among visual acuity, daily functional status, and mortality: the Salisbury eye evaluation study. JAMA Ophthalmol. (2014) 132:1400–6. doi: 10.1001/jamaophthalmol.2014.2847, PMID: 25144579 PMC7894742

[40] Knudtson MD. Age-related eye disease, visual impairment, and survival: the beaver dam eye study. Arch Ophthalmol. (2006) 124:243. doi: 10.1001/archopht.124.2.243, PMID: 16476894

[41] Jin S Trope GE Buys YM Badley EM Thavorn K Yan P . Reduced social participation among seniors with self-reported visual impairment and glaucoma. PLoS One. (2019) 14:e0218540. doi: 10.1371/journal.pone.0218540, PMID: 31335896 PMC6650048

[42] Global Health estimates 2019: Life expectancy, 2000–2019. Geneva: World Health Organization (2020).

[43] Zheng G Zhou B Fang Z Jing C Zhu S Liu M . Living alone and the risk of depressive symptoms: a cross-sectional and cohort analysis based on the China health and retirement longitudinal study. BMC Psychiatry. (2023) 23:853. doi: 10.1186/s12888-023-05370-y, PMID: 37978367 PMC10655346

[44] Steinmetz JD Bourne RRA Briant PS Flaxman SR Taylor HRB Jonas JB . Causes of blindness and vision impairment in 2020 and trends over 30 years, and prevalence of avoidable blindness in relation to VISION 2020: the right to sight: an analysis for the global burden of disease study. Lancet Glob Health. (2021) 9:e144–60. doi: 10.1016/S2214-109X(20)30489-7, PMID: 33275949 PMC7820391

