# Supplementary material for: Visual impairment and all-cause mortality: a real-world retrospective cohort study
Source: Front Public Health. 2025 Nov 13;13:1670906. doi: 10.3389/fpubh.2025.1670906 (PMC12657157; doi:10.3389/fpubh.2025.1670906)
Supplement: Supplementary file 1 [file Supplementary_file_1.docx]

**Supplementary Material**

Table S1. Characteristics of participants stratified by visual acuity category with follow-up time exceeding two years.

Table S2. Association between visual impairment and mortality.

Table S3. Association between visual impairment and mortality in female and male participants.

Table S4. Association between visual impairment and mortality in females and males stratified by age.

Table S5. The ICD-10 codes for chronic diseases and the corresponding diagnostic names.

| **Table S1. Characteristics of participants stratified by visual acuity category with follow-up time exceeding two years.** | | | | |
| --- | --- | --- | --- | --- |
|  | **Overall**  **(n = 158,700)** | **Non-VI group**  **(n = 119,045)** | **VI group**  **(n = 39,655)** | **SMD** |
| Age (year), mean ± SD | 60.59 (13.45) | 59.04 (13.62) | 65.25 (11.76) | 0.488 |
| Follow-up time (year), median [IQR] | 3.95 [3.12, 4.30] | 3.95 [3.11, 4.30] | 3.93 [3.15, 4.28] | 0.025 |
| Female, n (%) | 87,382 (55.06) | 65,121 (54.70) | 22,261 (56.14) | 0.029 |
| Marriage status, n (%) |  |  |  |  |
| Unmarried | 14,340 (9.04) | 99,15 (8.33) | 4,425 (11.16) | 0.098 |
| Married | 143,052 (90.14) | 108,201 (90.89) | 34,851 (87.89) |  |
| Education level, n (%) |  |  |  |  |
| Less than junior secondary | 124,453 (78.42) | 91,390 (76.77) | 33,063 (83.38) | 0.168 |
| More than junior secondary | 31,859 (20.07) | 25,819 (21.69) | 6,040 (15.23) |  |
| Urban residency status (%) |  |  |  |  |
| Rural | 81,819 (51.56) | 59,749 (50.19) | 22,070 (55.66) | 0.110 |
| Urban | 76,870 (48.44) | 59,291 (49.81) | 17,579 (44.33) |  |
| Drink status, n (%) |  |  |  |  |
| Never or quit | 122,230 (77.02) | 91,216 (76.62) | 31,014 (78.21) | 0.110 |
| Current drinker | 31,341 (19.75) | 23,445 (19.69) | 7,896 (19.91) |  |
| Smoke status, n (%) |  |  |  |  |
| Never or quit | 129,196 (81.41) | 96,325 (80.91) | 32,871 (82.89) | 0.109 |
| Current smoker | 24,075 (15.17) | 18,100 (15.2) | 5,975 (15.07) |  |
| BMI categories, n (%) |  |  |  |  |
| Normal weight | 80,690 (50.84) | 60,708 (51) | 19,982 (50.39) | 0.046 |
| Underweight | 5,508 (3.47) | 4,161 (3.50) | 1,347 (3.40) |  |
| Overweight | 49,928(31.46) | 37,106 (31.17) | 12,822 (32.33) |  |
| Obesity | 11,692 (7.37) | 8,634 (7.25) | 3,058 (7.71) |  |
| Diabetes, n (%) | 14,347 (9.04) | 10,362 (8.70) | 3,985 (10.05) |  |
| Hypertension, n (%) | 41,306 (26.03) | 29,919 (25.13) | 11387 (28.72) |  |
| BMI (kg/m^2^), mean ± SD | 6,233 (3.93) | 4,307 (3.62) | 1,926 (4.86) | 0.023 |
| SBP (mmHg), mean ± SD | 23.57 (3.06) | 23.55 (3.06) | 23.62 (3.08) | 0.199 |
| DBP (mmHg), median ± SD | 131.19 (17.07) | 130.33 (17.10) | 133.69 (16.75) | 0.048 |
| MABP (mmHg), mean ± SD | 78.50 (9.59) | 78.61 (9.72) | 78.16 (9.20) | 0.084 |
| Visual acuity categories were categorized according to presenting VA of the better eye (non-VI group, VA ≥ 0.5; VI group, VA < 0.5)  Data were reported as mean ± SD or median [IQR] for continuous variables and number (proportion) for categorical variables.  SMD was used to compare the variables between non-VI group and VI group.  Abbreviations: VI, visual impairment; VA, visual acuity; SMD, standard mean difference; SD, standard deviation; IQR, interquartile range; BMI, body mass index; SBP, systolic blood pressure; DBP, diastolic blood pressure; MABP, mean arterial blood pressure. | | | | |

| **Table S2. Association between visual impairment and mortality.** | | | | | | | | |  |
| --- | --- | --- | --- | --- | --- | --- | --- | --- | --- |
|  | **VA category** | **Follow-up**  **duration, PY** | **Event/Total, n** | **Mortality rate, 10^5^ PY** | **Model 1** | | | **Model 2** | |
|  |  |  |  |  | **HR (95% CI)** | ***p* value** | **HR (95% CI)** | | ***p* value** |
| All  (n = 158,700) | Non-VI group | 449,115.67 | 907/119,045 | 201.95 | Reference |  | Reference | |  |
|  | VI group | 148,711.50 | 610/39,655 | 410.19 | 1.20 (1.08–1.33) | < 0.001 | 1.22 (1.10–1.36) | | < 0.001 |
|  | Mild VI subgroup | 96,593.77 | 284/26,265 | 294.01 | 0.96 (0.84–1.10) | 0.5312 | 0.98 (0.86–1.12) | | 0.791 |
|  | Moderate VI subgroup | 48,243.04 | 301/12,377 | 623.92 | 1.53 (1.34–1.75) | < 0.001 | 1.55 (1.35–1.77) | | < 0.001 |
|  | Severe VI subgroup | 3,874.70 | 25/1,013 | 645.21 | 1.81 (1.22–2.70) | 0.004 | 1.75 (1.17–2.61) | | 0.006 |
|  | *p* for trend |  |  |  | 1.20 (1.13–1.28) | < 0.001 | 1.21 (1.14–1.29) | | < 0.001 |
| Old  (age ≥ 60 years)  (n = 94,497) | Non-VI group | 255,375.81 | 820/66,218 | 321.10 | Reference |  | Reference | |  |
|  | VI group | 107,256.25 | 590/28,279 | 550.08 | 1.21 (1.09–1.35) | < 0.001 | 1.23 (1.10–1.37) | | < 0.001 |
|  | Mild VI subgroup | 68,402.00 | 273/18,417 | 399.11 | 0.97 (0.84–1.11) | 0.646 | 0.99 (0.86–1.14) | | 0.878 |
|  | Moderate VI subgroup | 36,189.45 | 295/9,195 | 815.15 | 1.55 (1.35–1.77) | < 0.001 | 1.56 (1.36–1.79) | | < 0.001 |
|  | Severe VI subgroup | 2,664.80 | 22/667 | 825.58 | 1.67 (1.09–2.56) | 0.018 | 1.62 (1.06–2.48) | | 0.027 |
|  | *p* for trend |  |  |  | 1.21 (1.13–1.28) | < 0.001 | 1.21 (1.13–1.29) | | < 0.001 |
| Young to middle–aged  (age < 60 years)  (n = 64,203) | Non-VI group | 193,739.86 | 87/52,827 | 44.91 | Reference |  | Reference | |  |
|  | VI group | 41,455.25 | 20/11,376 | 48.24 | 0.87 (0.54–1.42) | 0.589 | 0.93 (0.57–1.52) | | 0.765 |
|  | Mild VI subgroup | 28,191.77 | 11/7,848 | 39.02 | 0.71 (0.38–1.34) | 0.290 | 0.78 (0.41–1.46) | | 0.431 |
|  | Moderate VI subgroup | 12,053.58 | 6/3,182 | 49.78 | 0.89 (0.39–2.03) | 0.779 | 0.89 (0.39–2.06) | | 0.792 |
|  | Severe VI subgroup | 1,209.90 | 3/346 | 247.95 | 4.43 (1.40–14.04) | 0.011 | 4.08 (1.28–13.03) | | 0.018 |
|  | *p* for trend |  |  |  | 1.05 (0.77–1.42) | 0.766 | 1.07 (0.79–1.45) | | 0.661 |
| Model 1: Adjusted for age and sex.  Model 2: Adjusted for age, sex, marital status, educational level, urban residency status, smoking status, drinking status, diabetes mellitus history, hypertension history, stroke history, BMI category, and MABP.  VA categories were categorized according to presenting VA of the better eye (non-VI group, VA ≥ 0.5; VI group, VA < 0.5; mild VI subgroup, 0.3 ≤ VA < 0.5; moderate VI subgroup, 0.1 ≤ VA < 0.3; severe VI subgroup, VA < 0.1).  Abbreviations: VI, visual impairment; VA, visual acuity; PY, person-year; HR, hazard ratio; CI, confidence interval; BMI, body mass index; MABP, mean arterial blood pressure. | | | | | | | | | |

| **Table S3. Association between visual impairment and mortality in female and male participants.** | | | | | | | | |  |
| --- | --- | --- | --- | --- | --- | --- | --- | --- | --- |
|  | **VA category** | **Follow-up**  **duration, PY** | **Event/Total, n** | **Mortality rate, 10^5^ PY** | **Model 1** | | | **Model 2** | |
|  |  |  |  |  | **HR (95% CI)** | ***p* value** | **HR (95% CI)** | | ***p* value** |
| Female  (n = 97,382) | Non-VI group | 246,691.22 | 368/65,121 | 149.17 | Reference | | Reference | | |
|  | VI group | 83,814.72 | 278/22,261 | 331.68 | 1.17 (1.00–1.37) | 0.056 | 1.21 (1.03–1.42) | | 0.022 |
|  | Mild VI subgroup | 53,178.74 | 126/14,391 | 236.94 | 0.92 (0.75–1.13) | 0.449 | 0.96 (0.78–1.18) | | 0.716 |
|  | Moderate VI subgroup | 28,704.85 | 140/7,352 | 487.72 | 1.45 (1.19–1.76) | < 0.001 | 1.48 (1.21–1.80) | | < 0.001 |
|  | Severe VI subgroup | 1,931.13 | 12/518 | 621.40 | 2.62 (1.47–4.67) | 0.001 | 2.51 (1.41–4.49) | | 0.002 |
|  | *p* for trend |  |  |  | < 0.001 |  | < 0.001 | |  |
| Male  (n = 71,318) | Non-VI group | 202,424.45 | 539/53,924 | 266.27 | Reference | | Reference | | |
|  | VI group | 64,896.78 | 332/17,394 | 511.58 | 1.23 (1.07–1.41) | 0.004 | 1.24 (1.08–1.43) | | 0.002 |
|  | Mild VI subgroup | 43,415.02 | 158/11,874 | 363.93 | 0.99 (0.82–1.18) | 0.880 | 1.00 (0.84–1.20) | | 0.964 |
|  | Moderate VI subgroup | 19,538.18 | 161/5,025 | 824.03 | 1.61 (1.35–1.93) | < 0.001 | 1.62 (1.35–1.94) | | < 0.001 |
|  | Severe VI subgroup | 1,943.58 | 13/495 | 668.87 | 1.43 (0.83–2.49) | 0.200 | 1.41 (0.81–2.45) | | 0.224 |
|  | *p* for trend |  |  |  | < 0.001 |  | < 0.001 | |  |
| Model 1: Adjusted for age and sex.  Model 2: Adjusted for age, sex, marital status, educational level, urban residency status, smoking status, drinking status, diabetes mellitus history, hypertension history, stroke history, BMI category, and MABP.  VA categories were categorized according to presenting VA of the better eye (non-VI group, VA ≥ 0.5, VI group, VA < 0.5; mild VI subgroup, 0.3 ≤ VA < 0.5; moderate VI subgroup, 0.1 ≤ VA < 0.3; severe VI subgroup, VA < 0.1).  Abbreviations: VI, visual impairment; VA, visual acuity; PY, person-year; HR, hazard ratio; CI, confidence interval; BMI, body mass index; MABP, mean arterial blood pressure. | | | | | | | | | |

| **Table S4. Association between visual impairment and mortality in females and males stratified by age.** | | | | | | | | | | |  |
| --- | --- | --- | --- | --- | --- | --- | --- | --- | --- | --- | --- |
|  |  | **VA category** | **Follow-up**  **duration, PY** | **Event/Total, n** | **Mortality rate, 10^5^ PY** | **Model 1** | | | **Model 2** | | |
|  |  |  |  |  |  | **HR (95% CI)** | ***p* value** | **HR (95% CI)** | | ***p* value** | |
| Old  (≥ 60 years) | Female  (n = 48,825) | Non-VI group | 129,490.73 | 328/33,457 | 253.30 | Reference |  | Reference | |  | |
|  |  | VI group | 58,586.95 | 272/15,368 | 464.27 | 1.21 (1.02–1.42) | 0.025 | 1.25 (1.06–1.47) | | 0.009 | |
|  |  | Mild VI subgroup | 36,725.32 | 123/9,830 | 334.92 | 0.96 (0.78–1.18) | 0.704 | 1.00 (0.81–1.24) | | 0.995 | |
|  |  | Moderate VI subgroup | 20,624.34 | 138/5,220 | 669.11 | 1.49 (1.21–1.82) | < 0.001 | 1.52 (1.24–1.86) | | < 0.001 | |
|  |  | Severe VI subgroup | 1,237.29 | 11/318 | 889.04 | 2.61 (1.43–4.76) | 0.002 | 2.53 (1.38–4.63) | | 0.003 | |
|  |  | *p* for trend |  |  |  | < 0.001 |  | < 0.001 | |  | |
|  | Male  (n = 45,672) | Non-VI group | 125,885.08 | 492/3,2761 | 390.83 | Reference |  | Reference | |  | |
|  |  | VI group | 48,669.30 | 318/12,911 | 653.39 | 1.22 (1.06–1.41) | 0.007 | 1.23 (1.06–1.42) | | 0.005 | |
|  |  | Mild VI subgroup | 31,676.68 | 150/8,587 | 473.53 | 0.98 (0.81–1.18) | 0.817 | 0.99 (0.82–1.19) | | 0.930 | |
|  |  | Moderate VI subgroup | 15,565.12 | 157/3,975 | 1008.67 | 1.60 (1.34–1.93) | < 0.001 | 1.61 (1.34–1.93) | | < 0.001 | |
|  |  | Severe VI subgroup | 1,427.50 | 11/349 | 770.58 | 1.26 (0.69–2.29) | 0.457 | 1.23 (0.67–2.24) | | 0.504 | |
|  |  | *P* for trend |  |  |  | < 0.001 |  | < 0.001 | |  | |
| Young to middle-aged  (< 60 years**)** | Female  (n = 38,557) | Non-VI group | 117,200.49 | 40/31,664 | 34.13 | Reference |  | Reference | |  | |
|  |  | VI group | 25,227.77 | 6/6,893 | 23.78 | 0.54 (0.23–1.27) | 0.159 | 0.55 (0.23–1.31) | | 0.178 | |
|  |  | Mild VI subgroup | 16,453.43 | 3/4,561 | 18.23 | 0.42 (0.13–1.35) | 0.144 | 0.43 (0.13–1.40) | | 0.162 | |
|  |  | Moderate VI subgroup | 8,080.51 | 2/2,132 | 24.75 | 0.55 (0.13–2.27) | 0.408 | 0.55 (0.13–2.31) | | 0.417 | |
|  |  | Severe VI subgroup | 693.83 | 1/200 | 144.13 | 3.48 (0.48–25.34) | 0.219 | 3.09 (0.42–22.68) | | 0.267 | |
|  |  | *p* for trend |  |  |  | 0.372 |  | 0.395 | |  | |
|  | Male  (n = 25,646) | Non-VI group | 76,539.37 | 47/21,163 | 61.41 | Reference |  | Reference | |  | |
|  |  | VI group | 16,227.48 | 14/4,483 | 86.27 | 1.18 (0.65–2.16) | 0.582 | 1.30 (0.71–2.38) | | 0.401 | |
|  |  | Mild VI subgroup | 11,738.34 | 8/3,287 | 68.15 | 0.97 (0.46–2.06) | 0.935 | 1.12 (0.52–2.40) | | 0.778 | |
|  |  | Moderate VI subgroup | 3,973.07 | 4/1,050 | 100.68 | 1.26 (0.45–3.50) | 0.662 | 1.23 (0.44–3.45) | | 0.700 | |
|  |  | Severe VI subgroup | 516.07 | 2/146 | 387.54 | 5.18 (1.25–21.41) | 0.023 | 4.96 (1.18–20.89) | | 0.029 | |
|  |  | *p* for trend |  |  |  | 0.222 |  | 0.176 | |  | |
| Model 1: Adjusted for age and sex.  Model 2: Adjusted for age, sex, marital status, educational level, urban residency status, smoking status, drinking status, diabetes mellitus history, hypertension history, stroke history, BMI category, and MABP.  VA categories were categorized according to presenting VA of the better eye (non-VI group, VA ≥ 0.5, VI group, VA < 0.5; mild VI subgroup, 0.3 ≤ VA < 0.5; moderate VI subgroup, 0.1 ≤ VA < 0.3; severe VI subgroup, VA < 0.1).  Abbreviations: VI, visual impairment; VA, visual acuity; PY, person-year; HR, hazard ratio; CI, confidence interval; BMI, body mass index; MABP, mean arterial blood pressure. | | | | | | | | | | | |

| **Table S5. The ICD-10 codes for chronic diseases and the corresponding diagnostic names.** | |
| --- | --- |
| **ICD-10 Code** | **Diagnostic Name** |
| Diabetes mellitus | |
| E10.000 | Type 1 diabetes mellitus with coma |
| E10.001 | Type 1 diabetes mellitus with hyperosmolarity coma |
| E10.100 | Type 1 diabetes mellitus with ketoacidosis |
| E10.101 | Type 1 diabetes mellitus with ketoacidotic coma |
| E10.102 | Type 1 diabetes mellitus with lactic acidosis |
| E10.200+ | Type 1 diabetes mellitus with kidney complications |
| E10.201 | Type 1 diabetes mellitus with nephropathy |
| E10.300 | Type 1 diabetes mellitus with ophthalmic complications |
| E10.301+ | Type 1 diabetes mellitus with retinopathy |
| E10.302+ | Type 1 diabetes mellitus with cataract |
| E10.400 | Type 1 diabetes mellitus with neurological complications |
| E10.401+ | Type 1 diabetes mellitus with peripheral neuropathy |
| E10.402+ | Type 1 diabetes mellitus with autonomic neuropathy |
| E10.403+ | Type 1 diabetes mellitus with neuropathy |
| E10.404+ | Type 1 diabetes mellitus with neurogenic bladder |
| E10.500 | Type 1 diabetes mellitus with peripheral circulatory complications |
| E10.501+ | Type 1 diabetes mellitus with peripheral angiopathy |
| E10.502+ | Type 1 diabetes mellitus with ischemic heart disease |
| E10.503+ | Type 1 diabetes mellitus with foot ulcer |
| E10.504 | Type 1 diabetes mellitus with gangrene |
| E10.600 | Type 1 diabetes mellitus with specified complications |
| E10.601 | Type 1 diabetes mellitus with mastopathy |
| E10.602 | Type 1 diabetes mellitus with growth retardation |
| E10.700 | Type 1 diabetes mellitus with multiple complications |
| E10.800 | Type 1 diabetes mellitus with unspecified complications |
| E10.900 | Type 1 diabetes mellitus |
| E10.901 | Latent autoimmune diabetes in adults |
| E11.000 | Type 2 diabetes mellitus with coma |
| E11.001 | Type 2 diabetes mellitus with hyperosmolar coma |
| E11.002 | Type 2 diabetes mellitus with hypoglycemic coma |
| E11.003 | Type 2 diabetes mellitus with hyperosmolar coma and ketoacidosis |
| E11.100 | Type 2 diabetes mellitus with ketoacidosis |
| E11.101 | Type 2 diabetes mellitus with lactic acidosis |
| E11.200+ | Type 2 diabetes mellitus with nephropathy |
| E11.300 | Type 2 diabetes mellitus with ophthalmic complications |
| E11.301+ | Type 2 diabetes mellitus with retinopathy |
| E11.400 | Type 2 diabetes mellitus with neurological complications |
| E11.401+ | Type 2 diabetes mellitus with peripheral neuropathy |
| E11.402+ | Type 2 diabetes mellitus with neurogenic bladder |
| E11.403+ | Type 2 diabetes mellitus with neuropathy |
| E11.404+ | Type 2 diabetes mellitus with autonomic neuropathy |
| E11.500 | Type 2 diabetes mellitus with peripheral circulatory complications |
| E11.501+ | Type 2 diabetes mellitus with peripheral angiopathy |
| E11.502 | Type 2 diabetes mellitus with gangrene |
| E11.600 | Type 2 diabetes mellitus with specified complications |
| E11.601 | Type 2 diabetes mellitus with foot ulcer |
| E11.700 | Type 2 diabetes mellitus with multiple complications |
| E11.800 | Type 2 diabetes mellitus with unspecified complications |
| E11.900 | Type 2 diabetes mellitus |
| E11.901 | Maturity onset diabetes of the young |
| E12.000 | Malnutrition-related diabetes mellitus with coma |
| E12.100 | Malnutrition-related diabetes mellitus with ketoacidosis |
| E12.200+ | Malnutrition-related diabetes mellitus with renal complications |
| E12.300+ | Malnutrition-related diabetes mellitus with ophthalmic complications |
| E12.400+ | Malnutrition-related diabetes mellitus with neurological complications |
| E12.500 | Malnutrition-related diabetes mellitus with peripheral circulatory complications |
| E12.600 | Malnutrition-related diabetes mellitus with specified complications |
| E12.700 | Malnutrition-related diabetes mellitus with multiple complications |
| E12.800 | Malnutrition-related diabetes mellitus with unspecified complications |
| E12.900 | Malnutrition-related diabetes mellitus without complications |
| E13.000 | Other specified diabetes mellitus with coma |
| E13.100 | Other specified diabetes mellitus with ketoacidosis |
| E13.101 | Secondary diabetes mellitus with ketoacidotic coma |
| E13.102 | Secondary diabetes mellitus with ketoacidosis |
| E13.200+ | Other specified diabetes mellitus with renal complications |
| E13.201+ | Lipodystrophic diabetes mellitus with nephropathy |
| E13.300+ | Other specified diabetes mellitus with ophthalmic complications |
| E13.400+ | Other specified diabetes mellitus with neurological complications |
| E13.500 | Other specified diabetes mellitus with peripheral circulatory complications |
| E13.600 | Other specified diabetes mellitus with specified complications |
| E13.700 | Other specified diabetes mellitus with multiple complications |
| E13.800 | Other specified diabetes mellitus with unspecified complications |
| E13.900 | Other specified diabetes mellitus |
| E13.901 | Hepatogenous diabetes |
| E13.902 | Mitochondrial diabetes |
| E13.903 | Steroid-induced diabetes |
| E13.904 | Lipodystrophic diabetes |
| E13.905 | Drug or chemical induced diabetes |
| E13.906 | Stress hyperglycemia |
| E13.907 | Secondary diabetes mellitus |
| E14.000 | Unspecified diabetes mellitus with coma |
| E14.100 | Unspecified diabetes mellitus with ketoacidosis |
| E14.200+ | Unspecified diabetes mellitus with renal complications |
| E14.300+ | Unspecified diabetes mellitus with ophthalmic complications |
| E14.400+ | Unspecified diabetes mellitus with neurological complications |
| E14.500 | Unspecified diabetes mellitus with peripheral circulatory complications |
| E14.600 | Unspecified diabetes mellitus with specified complications |
| E14.700 | Unspecified diabetes mellitus with multiple complications |
| E14.800 | Unspecified diabetes mellitus with unspecified complications |
| E14.900 | Unspecified diabetes mellitus |
| Hypertension | |
| I10.x00 | Essential (primary) hypertension |
| I10.x01 | Borderline hypertension |
| I10.x02 | Malignant hypertension |
| I10.x03 | Stage 1 hypertension |
| I10.x04 | Stage 2 hypertension |
| I10.x05 | Stage 3 hypertension |
| I10.x06 | Hypertensive emergency |
| I10.x07 | Benign hypertension |
| I10.x08 | Isolated systolic hypertension in the elderly |
| I10.x09 | Primary hypertension |
| I10.x10 | Hypertensive urgency |
| I10.x11 | Isolated systolic hypertension |
| I10.x12 | Resistant hypertension |
| I10.x13 | Low-renin hypertension |
| I10.x14 | Hypertensive sub-urgency |
| Stroke | |
| I60.000 | Subarachnoid hemorrhage from carotid siphon and bifurcation |
| I60.001 | Ruptured carotid artery aneurysm with subarachnoid hemorrhage |
| I60.100 | Subarachnoid hemorrhage from middle cerebral artery |
| I60.101 | Ruptured middle cerebral artery aneurysm with subarachnoid hemorrhage |
| I60.200 | Subarachnoid hemorrhage from anterior communicating artery |
| I60.201 | Ruptured anterior communicating artery aneurysm with subarachnoid hemorrhage |
| I60.300 | Subarachnoid hemorrhage from posterior communicating artery |
| I60.301 | Ruptured posterior communicating artery aneurysm with subarachnoid hemorrhage |
| I60.400 | Subarachnoid hemorrhage from basilar artery |
| I60.401 | Ruptured basilar artery aneurysm with subarachnoid hemorrhage |
| I60.500 | Subarachnoid hemorrhage from vertebral artery |
| I60.600 | Subarachnoid hemorrhage from specified intracranial arteries |
| I60.601 | Ruptured cerebellar artery aneurysm with subarachnoid hemorrhage |
| I60.700 | Subarachnoid hemorrhage from intracranial arteries |
| I60.701 | Ruptured intracranial artery aneurysm with subarachnoid hemorrhage |
| I60.800 | Subarachnoid hemorrhage, specified |
| I60.801 | Ruptured cerebral arteriovenous malformation with subarachnoid hemorrhage |
| I60.802 | Meningeal hemorrhage |
| I60.900 | Subarachnoid hemorrhage |
| I60.901 | Ruptured cerebral aneurysm |
| I60.902+ | Terson syndrome |
| I61.000 | Intracerebral hemorrhage in hemisphere, subcortical |
| I61.001 | Putaminal hemorrhage |
| I61.002 | Subcortical cerebral hemorrhage |
| I61.003 | Posterior cerebral artery hemorrhage |
| I61.004 | Basal ganglia hemorrhage |
| I61.005 | Internal capsule hemorrhage |
| I61.006 | External capsule hemorrhage |
| I61.100 | Cortical cerebral hemorrhage |
| I61.101 | Lobar hemorrhage |
| I61.200 | Hemispheric cerebral hemorrhage |
| I61.300 | Brain stem hemorrhage |
| I61.301 | Pontine hemorrhage |
| I61.400 | Cerebellar hemorrhage |
| I61.500 | Ventricular hemorrhage |
| I61.600 | Multiple cerebral hemorrhages |
| I61.800 | Cerebral hemorrhage, specified |
| I61.801 | Thalamic hemorrhage |
| I61.802 | Thalamus hemorrhage |
| I61.803 | Hypothalamic hemorrhage |
| I61.900 | Cerebral hemorrhage |
| I61.901 | Middle cerebral artery hemorrhage |
| I61.902 | Hypertensive cerebral hemorrhage |
| I61.903 | Cerebral hematoma |
| I61.904 | Hemorrhagic cerebral softening |
| I61.905 | Cerebrovascular rupture |
| I62.000 | Subdural hemorrhage |
| I62.001 | Subdural hematoma |
| I62.002 | Acute subdural hemorrhage |
| I62.003 | Chronic subdural hematoma |
| I62.100 | Non-traumatic extradural hemorrhage |
| I62.101 | Extradural hematoma |
| I62.900 | Intracranial hemorrhage |
| I63.000 | Cerebral infarction due to thrombosis of pre-cerebral arteries |
| I63.001 | Cerebral infarction due to thrombosis of basilar artery |
| I63.002 | Cerebral infarction due to thrombosis of carotid artery |
| I63.003 | Cerebral infarction due to thrombosis of vertebral artery |
| I63.100 | Cerebral infarction due to embolism of pre-cerebral arteries |
| I63.101 | Cerebral infarction due to embolism of basilar artery |
| I63.102 | Cerebral infarction due to embolism of carotid artery |
| I63.103 | Cerebral infarction due to embolism of vertebral artery |
| I63.200 | Cerebral infarction due to occlusion or stenosis of pre-cerebral arteries |
| I63.201 | Cerebral infarction due to stenosis of internal carotid artery |
| I63.202 | Cerebral infarction due to stenosis of common carotid artery |
| I63.203 | Cerebral infarction due to stenosis of carotid artery |
| I63.204 | Cerebral infarction due to occlusion of carotid artery |
| I63.205 | Cerebral infarction due to occlusion of basilar artery |
| I63.206 | Cerebral infarction due to stenosis of basilar artery |
| I63.207 | Cerebral infarction due to occlusion of vertebral artery |
| I63.208 | Cerebral infarction due to stenosis of vertebral artery |
| I63.300 | Cerebral infarction due to thrombosis of cerebral arteries |
| I63.301 | Thrombotic cerebral softening |
| I63.302 | Thrombotic hemiplegia |
| I63.400 | Cerebral infarction due to embolism of cerebral arteries |
| I63.401 | Embolic hemiplegia |
| I63.402 | Cerebral embolism |
| I63.500 | Cerebral infarction due to occlusion or stenosis of cerebral arteries |
| I63.501 | Cerebral infarction due to stenosis of cerebral arteries |
| I63.502 | Cerebral infarction due to occlusion of cerebral arteries |
| I63.600 | Cerebral infarction due to cerebral venous thrombosis |
| I63.800 | Cerebral infarction, specified |
| I63.801 | Lacunar infarction |
| I63.802 | Arteriosclerotic encephalopathy |
| I63.900 | Cerebral infarction |
| I63.901 | Brain stem infarction |
| I63.902 | Large-area cerebral infarction |
| I63.903 | Hemorrhagic |
| I63.904 | Cerebellar infarction |
| I63.905 | Multiple cerebral infarctions |
| I63.906 | Basal ganglia infarction |
| I63.907 | Thalamic infarction |
| I63.908 | Traumatic cerebral infarction |
| I64.x00 | Stroke, not specified as hemorrhage or infarction |
| I64.x01 | Cerebrovascular accident |
| I66.903 | Cerebral thrombosis |
| I69.000 | Sequelae of subarachnoid hemorrhage |
| I69.100 | Sequelae of intracerebral hemorrhage |
| I69.200 | Sequelae of other non-traumatic intracranial hemorrhage |
| I69.300 | Sequelae of cerebral infarction |
| I69.400 | Sequelae of stroke, not specified as hemorrhage or infarction |
| I69.800 | Sequelae of specified cerebrovascular diseases |
| I69.801 | Sequelae of cerebral thrombosis |
| I69.802 | Sequelae of cerebrovascular disease |
| Heart attack | |
| I21.000 | Acute ST Elevation Myocardial Infarction of Anterior Wall |
| I21.001 | Acute ST Elevation Myocardial Infarction of Anteroseptal Wall |
| I21.002 | Acute ST Elevation Myocardial Infarction of Anterolateral Wall |
| I21.003 | Acute ST Elevation Myocardial Infarction of Extensive Anterior Wall |
| I21.004 | Acute Anterior Wall Myocardial Infarction |
| I21.005 | Acute Anteroseptal Myocardial Infarction |
| I21.006 | Acute Anterolateral Myocardial Infarction |
| I21.007 | Acute Extensive Anterior Wall Myocardial Infarction |
| I21.100 | Acute ST Elevation Myocardial Infarction of Inferior Wall |
| I21.101 | Acute ST Elevation Myocardial Infarction of Inferoseptal Wall |
| I21.102 | Acute ST Elevation Myocardial Infarction of Inferolateral Wall |
| I21.103 | Acute Inferior Wall Myocardial Infarction |
| I21.104 | Acute Inferoseptal Myocardial Infarction |
| I21.105 | Acute Inferolateral Myocardial Infarction |
| I21.200 | Acute Transmural Myocardial Infarction of Specified Site |
| I21.201 | Acute ST Elevation Myocardial Infarction of High Lateral Wall |
| I21.202 | Acute ST Elevation Myocardial Infarction of True Posterior Wall |
| I21.203 | Acute ST Elevation Myocardial Infarction of Right Ventricle |
| I21.204 | Acute High Lateral Wall Myocardial Infarction |
| I21.205 | Acute True Posterior Wall Myocardial Infarction |
| I21.206 | Acute Right Ventricular Myocardial Infarction |
| I21.207 | Acute Inferior Wall Right Ventricular Myocardial Infarction |
| I21.208 | Acute Inferior Wall True Posterior Wall Myocardial Infarction |
| I21.210 | Acute Lateral Wall Myocardial Infarction |
| I21.211 | Acute Anterior Wall Inferior Wall Myocardial Infarction |
| I21.212 | Acute Inferior Wall Lateral Wall True Posterior Wall Myocardial Infarction |
| I21.300 | Acute Transmural Myocardial Infarction |
| I21.301 | Acute ST Elevation Myocardial Infarction |
| I21.302 | Post Coronary Artery Bypass Graft Myocardial Infarction |
| I21.303 | Post Percutaneous Coronary Intervention Myocardial Infarction |
| I21.400 | Acute Subendocardial Myocardial Infarction |
| I21.401 | Acute Non-ST Elevation Myocardial Infarction |
| I21.900 | Acute Myocardial Infarction |
| I21.901 | Coronary Artery Rupture |
| I21.902 | Acute Multivessel Myocardial Infarction |
| I22.000 | Subsequent Myocardial Infarction of Anterior Wall |
| I22.100 | Subsequent Myocardial Infarction of Inferior Wall |
| I22.800 | Subsequent Myocardial Infarction of Specified Site |
| I22.900 | Acute Recurrent Myocardial Infarction |
| I23.000 | Hemopericardium as a Complication of Acute Myocardial Infarction |
| I23.100 | Atrial Septal Defect as a Complication of Acute Myocardial Infarction |
| I23.200 | Ventricular Septal Rupture as a Complication of Acute Myocardial Infarction |
| I23.300 | Cardiac Rupture as a Complication of Acute Myocardial Infarction |
| I23.400 | Tendon Rupture as a Complication of Acute Myocardial Infarction |
| I23.500 | Papillary Muscle Rupture as a Complication of Acute Myocardial Infarction |
| I23.600 | Thrombosis of Atrium, Auricle, and Ventricle as an Early Complication of Acute Myocardial Infarction |
| I23.601 | Ventricular Mural Thrombosis as a Complication of Acute Myocardial Infarction |
| I23.800 | Specified Early Complications of Acute Myocardial Infarction |
| I25.200 | Old Myocardial Infarction |
| I25.201 | Old Lateral Wall Myocardial Infarction |
| I25.202 | Old Posterior Wall Myocardial Infarction |
| I25.203 | Old Anterior Wall Myocardial Infarction |
| I25.204 | Old Anteroseptal Myocardial Infarction |
| I25.205 | Old Inferior Wall Posterior Wall Myocardial Infarction |
| I25.206 | Old Inferior Wall Anterior Wall Myocardial Infarction |
| I25.207 | Old Inferior Wall Myocardial Infarction |
| I25.208 | Old Inferior Wall True Posterior Wall Myocardial Infarction |
